# Public health messages on arboviruses transmitted by *Aedes aegypti* in Brazil

**DOI:** 10.1186/s12889-021-11339-x

**Published:** 2021-07-09

**Authors:** India L. Clancy, Robert T. Jones, Grace M. Power, James G. Logan, Jorge Alberto Bernstein Iriart, Eduardo Massad, John Kinsman

**Affiliations:** 1 Department of Public Health, Environments & Society, London School of Hygiene & Tropical Medicine, London, UK; 2 Department of Disease Control, London School of Hygiene & Tropical Medicine, London, UK; 3 MRC Integrative Epidemiology Unit, Population Health Sciences, Bristol Medical School, University of Bristol, Bristol, UK; 4 Institute of Collective Health, Universidade Federal da Bahia, Salvador, Brazil; 5 School of Applied Mathematics, Fundacao Getulio Vargas, Rua Praia de Botafogo 190, Rio de Janeiro, RJ CEP 22250-900 Brazil; 6 Department of Epidemiology and Global Health, Faculty of Medicine, Umeå University, Umeå, Sweden

**Keywords:** Arbovirus, Zika, Poster, Health communication, Public health messages, Health belief model

## Abstract

**Background:**

The outbreak of Zika virus in Brazil in 2015 followed the arrival of chikungunya in 2014 and a long history of dengue circulation. Vital to the response to these outbreaks of mosquito-borne pathogens has been the dissemination of public health messages, including those promoted through risk communication posters. This study explores the content of a sample of posters circulated in Brazil towards the end of the Zika epidemic in 2017 and analyses their potential effectiveness in inducing behaviour change.

**Methods:**

A content analysis was performed on 37 posters produced in Brazil to address outbreaks of mosquito-borne pathogens. The six variables of the Health Belief Model were used to assess the potential effectiveness of the posters to induce behaviour change.

**Results:**

Three overarching key messages emerged from the posters. These included (i) the arboviruses and their outcomes, (ii) a battle against the mosquito, and (iii) a responsibility to protect and prevent. Among the six variables utilised through the Health Belief Model, cues to action were most commonly featured, whilst the perceived benefits of engaging in behaviours to prevent arbovirus transmission were the least commonly featured.

**Conclusions:**

The posters largely focused on mosquito-borne transmission and the need to eliminate breeding sites, and neglected the risk of the sexual and congenital transmission of Zika and the importance of alternative preventive actions. This, we argue, may have limited the potential effectiveness of these posters to induce behaviour change.

**Supplementary Information:**

The online version contains supplementary material available at 10.1186/s12889-021-11339-x.

## Background

Arboviruses have long posed a threat to public health in Brazil. An outbreak of dengue virus (DENV) was reported in 1845, and continuous reintroductions have led to the hyperendemic circulation of all four DENV serotypes. Chikungunya (CHIKV) was first documented in Brazil in 2014 and was in circulation when the Zika virus (ZIKV) reached South America in 2015 [1, 2]. By the end of 2016, an additional outbreak of yellow fever (YFV) emerged in Brazil [3].

These viruses are primarily transmitted by *Aedes aegypti* [4]. *Aedes* mosquitoes are associated with human habitation and tend to breed in artificial containers such as buckets, flower pots and tyres within and in close proximity to human habitations [5]. Vector control strategies, therefore, include the elimination of stagnant water in these containers to reduce mosquito breeding sites [6]. In addition to being transmitted by mosquitoes, ZIKV can also be transmitted by sexual intercourse, vertically from an infected mother to her foetus, and via blood transfusion [7]. ZIKV is responsible for a set of conditions now known as congenital Zika syndrome (CZS), which include microcephaly and serious brain anomalies in infants [8]. As our knowledge regarding these complications evolved, the Brazilian Ministry of Health recognised the importance of intensifying actions to prevent ZIKV transmission )[10]. This included the establishment of risk communication strategies to educate the public about tackling the mosquitoes that transmit the virus [10].

Risk communication forms an integral component of the response to any public health emergency. When designed effectively, the dissemination of information, that in turn influences the uptake of behaviours, can protect the health of individuals and their communities [9]. Whilst planning these strategies, the Brazilian Ministry of Health’s overarching goal was to be able to communicate risks to the general public effectively, without inducing alarm [10]. By the end of November 2015, renewed campaigns against *Ae. aegypti* were emerging to warn the general public about ZIKV transmission, and in January 2016 the high-profile ZikaZero campaign was launched, which promoted the widespread knowledge of basic actions for individuals to protect themselves against the mosquito [10].

Here, we review the content and potential effectiveness of public health messages on arbovirus outbreaks conveyed to the Brazilian population, by examining the textual and visual content of posters photographed towards the end of the ZIKV outbreak. We use the six key variables of the Health Belief Model (HBM) to consider the potential for behaviour change in individuals.

The variables of the HBM are *perceived susceptibility* (the degree to which an individual believes that they are vulnerable to developing a health condition), *perceived severity* (an individual’s perceptions regarding the seriousness of the condition and the subsequent health impacts that can arise from it), *perceived benefits* (an individual’s assessment of the value of engaging in a health-promoting behaviour to decrease risk of disease), *perceived barriers* (an individual’s assessment of the obstacles to behaviour change), *self-efficacy* (an individual’s perceptions regarding their ability to engage in preventive behaviours), and *cues to action* (the existence of prompts for individuals to engage in a preventive behaviour). The model has been widely used in the production of effective and successful public health messaging. For example, the HBM was used to develop educational interventions to encourage breast cancer screening [11] and materials to promote weight-management [12]. It has also been employed as a tool for investigating health behaviour determinants, such as those that affect regular dental attendance among primary school-aged children [13]. The HBM was selected here because of its value in predicting the uptake of preventive health behaviours [14, 15], which can provide insight into the potential effectiveness of public health messages for ZIKV.

To our knowledge, there is no published literature on the content and analysis of posters containing public health messages on arboviruses transmitted by *Aedes* mosquitoes specifically in Brazil. Our study contributes to the strengthening of arbovirus outbreak preparedness by identifying opportunities for inducing engagement in preventive behaviours, and providing recommendations for public health messages and risk communication in the wake of a future epidemic.

## Methods

### Data collection methods and sampling

Between April and August 2017, forty-three public health messaging posters were photographed across communities in Brazil including within Salvador and São Paulo. Sampling was opportunistic rather than systematic: all relevant posters seen were photographed by the research team. The posters are presented in Supplementary materials 1 and 2 with English translations.

### Data cleaning

Of the forty-three posters, three were identified as duplicates and these were excluded from the analysis. A further three posters were identified as being misclassified as standalone posters; instead, each of them formed a second page to other posters within the sample. These posters were analysed alongside their paired pages. The set of thirty-seven complete posters can be divided into three categories: national-level messages (6/37, posters denoted ‘N’), Salvador (24/37, denoted ‘S’), and São Paulo (7/37, denoted ‘SP’).

### Data analysis

A content analysis was conducted, involving the systematic review of the images and text of the photographed posters using assigned codes (labels) to indicate the presence of specific content. All posters were translated from Brazilian Portuguese to English prior to coding. Translations were provided by a native Brazilian Portuguese speaker, to ensure the accuracy of these translations and their interpretations in the English language.

The posters were imported into NVivo 12 (QSR International) for coding of both textual and visual data. First, inductive coding was used by reviewing each poster to identify coding categories until a point of saturation was reached. For example, “cover/wash buckets and containers” and “empty/turn bottles upside down” were inductive codes, and each poster was scored for the presence or absence of these two codes.

Deductive coding was then employed, using pre-defined codes that are representative of the variables of the HBM [14, 16]. The codes were adapted to the context of arbovirus-related preventive behaviours (Table 1). Once coding was complete, codes with similar themes were organised into higher categories. To form a higher category, a word or phrase was identified that described a group of the codes. For example, “elimination of breeding sites” was selected as a higher category for the two codes listed above. These higher categories were formed to identify overarching, key messages from the data.

**Table 1:** Definitions used for the six major variables of the HBM

**Perceived Susceptibility**	Reference to personal risk of contracting the disease if action is not taken to prevent transmission. For example: use of the personal pronouns ‘you’ and ‘us’ to emphasise personal risk.
**Perceived Severity**	Reference to any serious outcomes of not taking action to prevent transmission. For example: mention of the severity of symptoms or the potential risk of fatality for microcephaly in babies.
**Perceived Benefits**	Reference to any positive outcomes of taking action to prevent transmission. For example: protecting family and friends or the avoidance of serious health outcomes.
**Perceived Barriers**	Reference to addressing any of the barriers that may exist with regard to taking action to prevent transmission. For example: complex or time-consuming preventive actions.
**Self-Efficacy**	Reference to highlighting the competence or ability of the individual to take action to prevent transmission. For example: emphasising the integral nature of the individual in preventing arbovirus transmission.
**Cues to Action**	Inclusion of any trigger that prompts the individual to engage in taking action to prevent transmission. For example: if the poster provides instructions or reminders of specific preventive actions to take, a place to go to, such as a hospital for medical help after developing symptoms, or the provision of a telephone number to call.

## Results

The content analysis resulted in the emergence of forty-eight coding categories, of which, forty-two were inductivelyidentified. In addition, six deductively-identified coding variables, representative of the HBM, were present. All posters (37/37) featured written text, and 95% (35/37) of the posters featured at least one image.

Following the coding of the posters and the organisation of these codes into higher categories, three overarching, key messages emerged from the content analysis. These were: (i) the arboviruses and their outcomes, (ii) a battle against the mosquito, and (iii) a responsibility to protect and prevent.

### The arboviruses and their outcomes

**Fig. 1:**
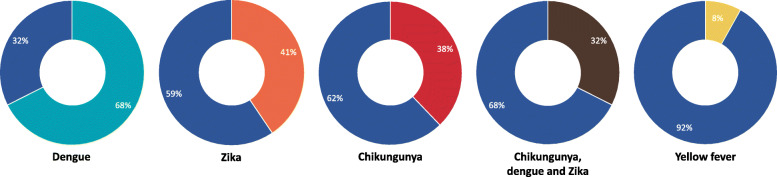
Proportion of all posters conveying information relating to each arbovirus

Information regarding the nature of the various arboviruses, and their outcomes, contributed to a significant proportion of the messages across the posters. The majority (68%, 25/37) of the posters conveyed information relating to DENV, whilst 41% (15/37) of the posters mentioned ZIKV and 38% (14/37) mentioned CHIKV (Fig. 1). Approximately one third (32%, 12/37) of the posters discussed all three of these arboviruses. YFV was mentioned in 8% (3/37) of the posters.

#### Disease outcomes

**Fig. 2:**
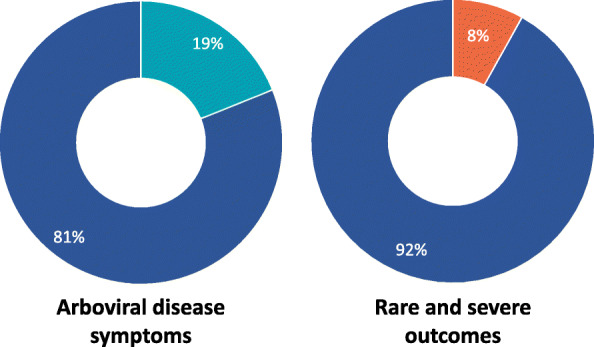
Proportion of all posters conveying information relating to disease outcomes

Arboviral disease symptoms were described in 19% (7/37) of the posters (Fig. 2). Several of these utilised diagrams of the human body to illustrate the variety of symptoms that an individual may develop. Further, discussion of the rare and severe outcomes of ZIKV were identified in 8% (3/37) of the posters, with 5% (2/37) discussing microcephaly and 3% (1/37) discussing Guillain-Barré syndrome.

#### Modes of transmission

**Fig. 3:**
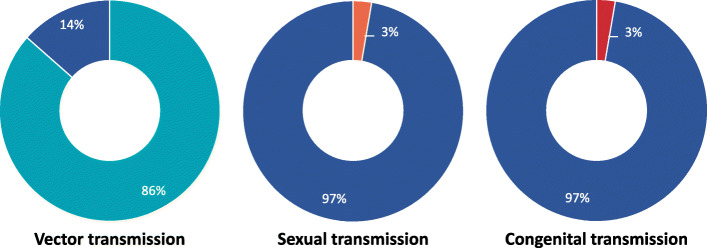
Proportion of all posters conveying information relating to each mode of transmission

The discussion of the modes of transmission of the arboviruses were heavily focused on the role of the mosquito in vector transmission: 86% (32/37) of the posters contained references to either mosquitoes in general (62%, 23/37), or more specifically, the *Ae. aegpyti* mosquito (24%, 9/37). By contrast, 3% (1/37) of the posters referred to the sexual transmission of ZIKV. A further 3% (1/37) of the posters referred to the congenital transmission of ZIKV (Fig. 3).

### A Battle against the mosquito

The framing of the prevention of the transmission of the arboviruses as a battle against the mosquito was a second overarching, key message that was identified across the posters.

#### The challenge of the mosquito

Many of the poster’s messages indicated the need to overcome the challenge of the mosquito, and typically utilised language that posited the *Ae. aegypti* mosquito as an enemy to the population. For example, two (5%, 2/37) of the posters referred to the potential for the mosquito to ‘attack’ the individual. Further references were made to a ‘battle’, a ‘fight’ or a ‘war’, predominantly communicated through slogans such as: ‘*Risk has risen and the battle needs to be even tougher.*’ (Poster number S1), ‘*Periodically fight against the mosquito.*’ (S14), and ‘*The war continues*’ (N4). Another slogan that exhibited this challenge of the mosquito was utilised by a campaign circulated in Salvador by the state of Bahia, which stated: ‘*Now it’s everyone against the mosquito.*’ (S1). This slogan was also accompanied by a logo, where the mosquito was visually presented as being a target.

This overarching message of the challenge of the mosquito persisted across many of the images featured. Of the twenty-nine images and logos of mosquitoes across the thirty-seven posters, fourteen (48%) consisted of a prohibited sign (a red circle with a red diagonal line through it) overlying the image of the mosquito. These were often paired with statements relating to opposition of the mosquito.

#### Preventive actions

The elimination of mosquito breeding sites formed the predominant focus of the preventive actions that individuals could take to prevent the transmission of the arboviruses: 62% (23/37) of the posters referred to breeding sites or their elimination. References to the elimination of stagnant water were common, present in 54% (20/37) of the posters. Many of these posters (46%, 17/37) warned individuals about accumulating water and prompted them to either cover, wash, or empty household objects that may collect water inside of them.

Mention of preventive actions that are unrelated to breeding sites were limited, but largely focused on prevention through the avoidance of mosquito bites. These included instructions regarding the use of repellents and mosquito nets. Two modes of disease prevention identified in the posters that did not relate to the control of the mosquito population or prevention of bites were vaccination against YFV, and the use of contraceptives, which prevent sexual transmission of ZIKV (Table 2).

**Table 2:** Preventive actions featured

Preventive Action	Number of Posters	Proportion of posters (%)
Elimination of Breeding Sites	23	62
Rubbish Clearance	12	32
Cover/Wash Buckets and Containers	16	43
Empty/Turn Bottles Upside Down	9	24
Empty/Wash Plant Pots	9	24
Gutter/Drain Clearance	6	16
Empty/Cover Tyres	6	16
Water Treatment	3	8
Clean Air Conditioning	2	5
Cover Toilets	2	5
Use of Mosquito Nets	2	5
Use of Repellents	1	3
Vaccination (YFV)	2	5
Use of Contraceptives	1	3

**Fig. 4:**
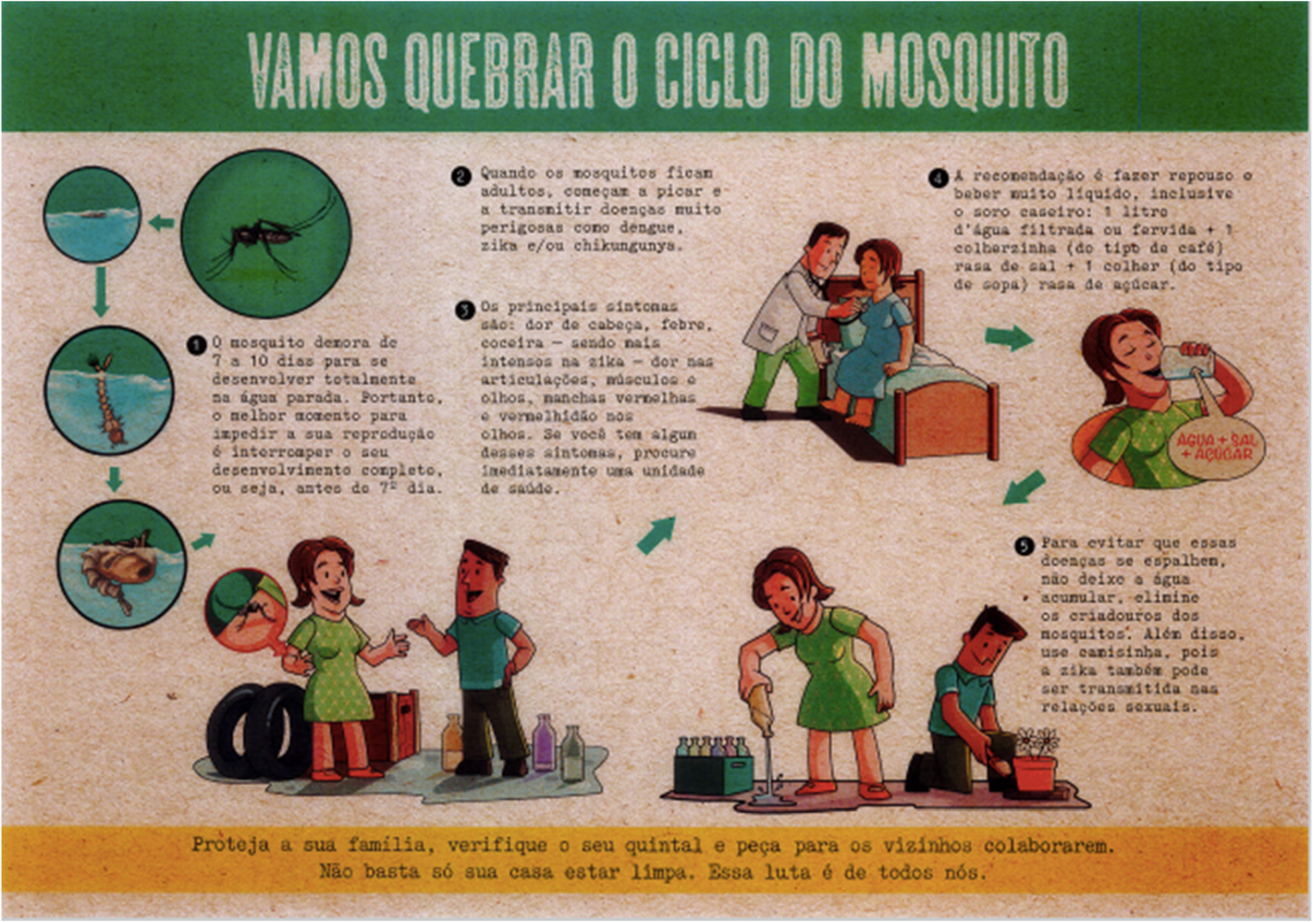
Poster displaying preventive measures against ZIKV. The text at point 5 translates to English as: ‘To prevent these diseases from spreading, do not let water accumulate, eliminate mosquito breeding grounds. Also, use a condom, as Zika can also be transmitted during sex.’ (N1)

Images referring to preventive actions were present in 49% (18/37) of the posters. These images included small, visual representations of the preventive actions being carried out, in addition to images of the household objects that the actions being described were related to (Fig. 4).

### A responsibility to protect and prevent

A further, overarching message that emerged from the content analysis was for individuals to take responsibility to protect themselves and those around them, and to prevent the transmission of arboviruses.

#### Responsibility for actions

Amongst the posters, the message of responsibility encompassed the role of the individual, the role of others, and collective action. However, this responsibility was disproportionately placed on the individual, with 59% (22/37) of the posters containing messages pertaining to personal responsibility. These included messages such as: ‘*Dengue has no vaccine, the vaccine is your action.*’ (SP5), and ‘*Don’t breed the mosquito for it to attack you.*’ (S17).

The importance of the actions of others was highlighted in a comparatively smaller proportion of the posters: 5% (2/37) of posters conveyed the message that the individual was responsible for inducing the actions of others. The examples identified were: ‘*Take care of your home, mobilize your family, your neighbours and your community.*’ (Poster N6) and ‘*Protect your family, check your yard and ask the neighbours to cooperate.*’ (N1).

References to the importance of collective action were present in 41% (15/37) of the posters. These messages were conveyed primarily through slogans, the most frequently used of which were: ‘*Everyone against Aedes aegypti*!’ (SP3, S19, S20 & S21), and ‘*A mosquito is not stronger than an entire country.*’ (SP2, S14 & N6). This latter slogan dominated Brazil’s ZikaZero campaign [17]

#### Personal and community protection

Messages pertaining to an urgency to provide protection against the arboviruses were also found to be prevalent amongst the posters: 27% (10/37) of the posters referred to the need to provide personal protection, and 22% (8/37) of the posters referred to the need to protect family members. Only one poster (3%) provided a specific mention of the need to protect babies or pregnant women. Furthermore, 16% (6/37) of the posters referred to the need to take action to protect neighbours or the community.

### Presence of the health belief model variables

At least one of the six variables of the HBM were identified across 97% (36/37) of the posters, but none of the posters contained all six variables (Table 3).

**Table 3:** Number of posters by number of variables present

Number of Variables Present	Number of Posters	Proportion of posters (%)
0	1	3
1	9	24
2	15	41
3	6	16
4	4	11
5	2	5
6	0	0

Cues to action were the most predominantly featured variable of the HBM, present in 84% (32/37) of the posters. The perceived benefits of taking action to prevent the transmission of arboviruses were the least commonly featured, present in 11% (4/37) of the posters (Table 4).

**Table 4:** Number of posters by variable

Variable	Number of Posters	Proportion of posters (%)
**Cues to Action**	31	84
**Perceived Barriers**	16	43
**Perceived Severity**	13	35
**Perceived Susceptibility**	12	32
**Self-Efficacy**	11	30
**Perceived Benefits**	4	11

#### Perceived susceptibility

Messages conveying perceived susceptibility emphasised the personal risk of becoming unwell, should individuals fail to take action. Two posters contained the text: ‘*Did you know that the chance of getting sick with dengue inside your house is much higher than in the street? Studies leave no doubt: more than 90% of the breeding sites are in houses. If you don’t prevent it, you can turn out to be the first target of the mosquito.*’ (S7 & S17).

**Fig. 5:**
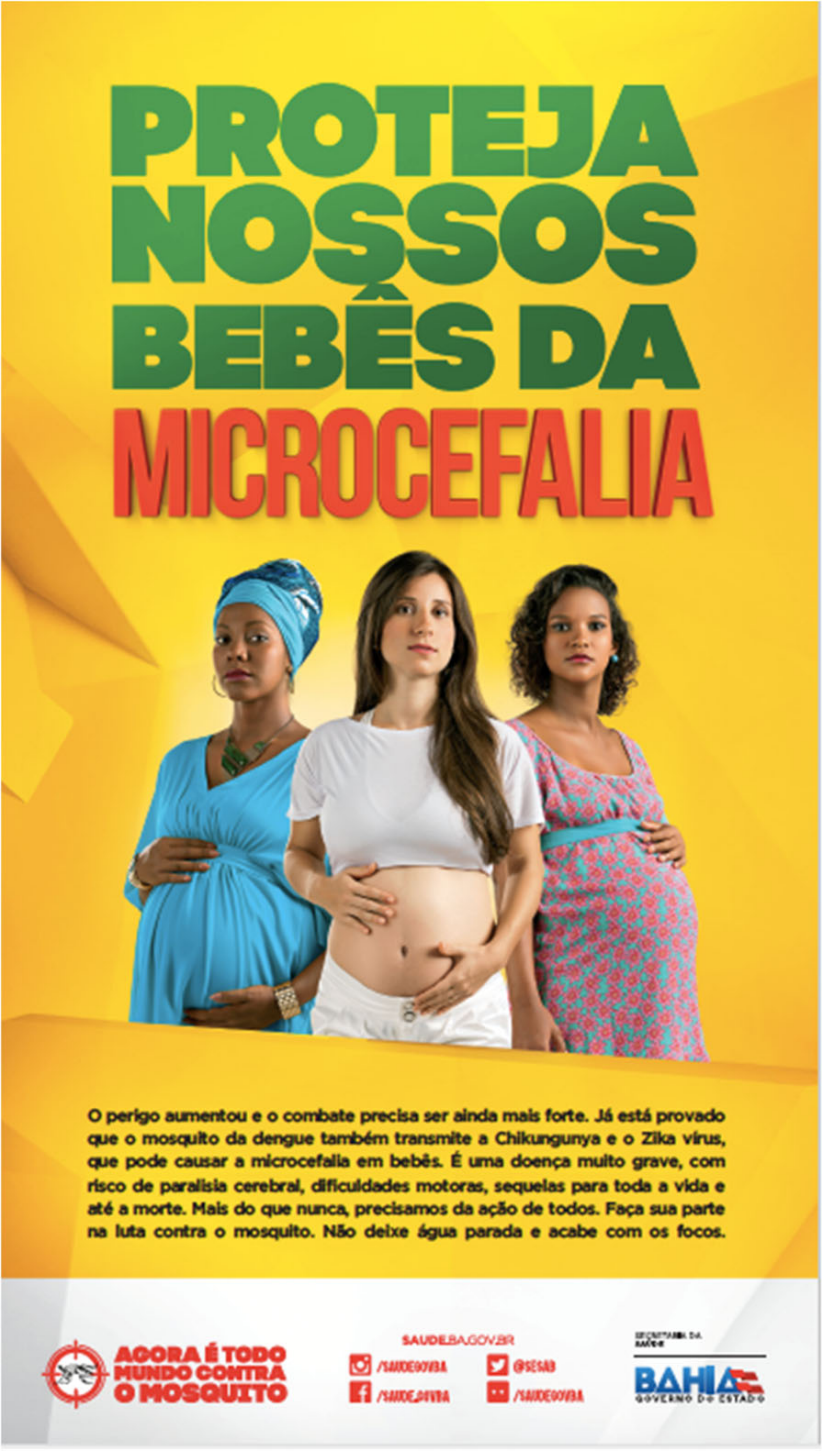
Poster from Salvador with images supporting the text, which highlights the link between ZIKV infection and microcephaly in the baby (S1)

Furthermore, the susceptibility was conveyed through images. Three (8%, 3/37) of the posters contained images that were identified as conveying this susceptibility, such as to pregnant women (Fig. 5), a family, and a diverse group of Brazilian citizens.

#### Perceived severity

Messages that conveyed the perceived severity of the health outcomes of arbovirus infection were also commonly identified. This included reference to the severity of symptoms, with posters referring to arboviruses as being ‘dangerous’, ‘severe’ or inducing ‘serious illness’. Three (8%, 3/37) of the posters detailed the seriousness of the severe conditions that can be developed as a result of ZIKV infection, and one poster (3%, 1/37) contained an image that was coded for perceived severity. This poster depicted the *Ae. aegypti* mosquito as a Grim Reaper figure, a symbol of death.

#### Perceived benefits

Messages coded for perceived benefits included the mention of the positive outcomes of taking action to prevent the transmission of arboviruses, conveyed primarily through a focus on the importance of engaging in preventive behaviours to protect others. For example, two posters obtained in Salvador stated: ‘*Pay attention, in order to finish up with this threat, take responsibility. For Bahia, for your city, for your neighbourhood and mainly for you and your family.*’ (S7 & S17).

#### Perceived barriers

None of the posters were identified as containing messages that specifically referred to the existence of barriers to preventing the transmission of arboviruses. However, 43% (16/37) of the posters were identified as indicating ways to overcome such potential barriers. These included the provision of alternative actions to take to eliminate household breeding sites, if it were not possible to complete the initial instructions given. For example, two posters provided instructions with these alternatives: ‘*Fill plates of flower vases with sand up to the top. Another option for the plates for plants is to wash them weekly.*’ (S14, S24), thereby offering an option for those who do not have access to sand for their vases as a way of preventing mosquito larvae from developing.

Additionally, 8% (3/37) of the posters emphasised the simplicity of actions that can be taken to prevent the transmission of arboviruses, tackling presumptions of the complex and time-consuming nature of these actions. For example, one poster stated: ‘*Simple actions can prevent Aedes aegypti’s proliferation.*’ (S16).

Approximately one third (32%, 12/37) of the posters were identified as containing images that provided a visual representation of the actions being described, such as emptying water containers or clearing roof gutters, demonstrating what the individual needs to do to complete the action.

#### Self-efficacy

Messages that conveyed self-efficacy emphasised the ability of the individual to tackle the mosquito, should they decide to take action. In one such example, the poster stated: ‘*When you take responsibility, the one that dies is the dengue mosquito’* (S5). Of the posters studied, 11% (4/37) also highlighted the integral nature of the individual in the national fight against the mosquito: ‘*Everyone needs to take action now more than ever. Do your part in this fight against the mosquito.*’ (S1).

#### Cues to action

Posters identified as including cues to action predominantly provided instructions for actions to eliminate breeding sites, focusing on the elimination of stagnant water. The cues to action identified varied in their instructional depth. Several posters warned individuals about the presence of stagnant water, or encouraged its elimination, but offered no instructions, while other posters provided detailed instructions or a checklist of actions to take to remove sources of stagnant water.

In addition to the cues focusing on breeding site elimination, 22% (8/37) of the posters contained instructions urging individuals to seek medical help if they developed the symptoms of arboviral infection, or to be vaccinated against YFV. As an example, when referring to the symptoms of ZIKV infection, one poster stated: ‘*If you experience any of these symptoms, drink plenty of water and seek a health centre. If even after the care you continue with severe pain in the belly and vomit, immediately return to a health unit.*’ (N3). Almost half (46%, 17/37) of the posters directed readers to a website to gain further information about the arboviruses, or provided a telephone number to report the presence of a breeding site.

## Discussion

The content analysis revealed the emergence of three overarching, key messages from the posters: information regarding the arboviruses and their outcomes, a battle against the mosquito and a responsibility to protect and prevent.

### The arboviruses and their outcomes

In the 5 months leading to 31st August 2017, when the posters analysed in this study were photographed, nationwide confirmed autochthonous cases of ZIKV increased from 131,643 to 137,288. No further cases were reported that year [18], indicating that the posters were captured towards the end of the ZIKV outbreak. Meanwhile, cases of DENV peaked in April 2017 and reached a total of 252,054 cases by the end of the year [19, 20]. Perhaps as a consequence of this timing and difference in disease burden, DENV was the most commonly featured arbovirus across the posters, followed by ZIKV. CHIKV was also featured on more than one third of the posters, and was responsible for 195,962 disease cases in Brazil that year [21]. However, few of the posters referred to the YFV, despite an outbreak beginning in late 2016. Between 6th January and 16th March 2017, Brazil reported 933 suspected and 424 confirmed cases of YFV, including 249 deaths (112 suspected and 137 confirmed) [22]. It should be noted that the majority of the posters were obtained in Salvador, a northeast region, while the YFV outbreak predominantly occurred across the southeast of Brazil [23]. Nonetheless, large parts of Brazil either reported cases of YFV or were at risk for YFV transmission [22], and this lack of posters raises the question of whether the risks posed by the YFV were adequately communicated to the Brazilian population.

An emphasis was overwhelmingly placed on the role of the mosquito as the vector of transmission, which is not unexpected given the focus on DENV combined with the relative importance of mosquito transmission for ZIKV compared to other modes of transmission [24]. However, with 41% (15/37) of the posters referring to ZIKV, it is concerning that there was just a single mention of each congenital or sexual transmission of the virus. Evidence for the association between ZIKV and sexual transmission, in addition to transmission from the mother to the foetus, was increasingly well documented by the late stage of the epidemic when the posters were photographed [25, 26]. Indeed, in early 2016 the US CDC recommended that men with a pregnant partner should use a condom or abstain from sex for the duration of pregnancy if they live in an area where ZIKV was transmitted, because of the concern of microcephaly in infected newborns [27]. Health information campaigns at the time were, therefore, expected to advise the public to avoid ZIKV infection through exposure to mosquitoes and unprotected sex [28], and in October 2016 Althaus & Low [29] called for information and advice for the public to give clear messages about the relative contributions of mosquito-borne, vertical, sexual, and bloodborne transmission. Given that the posters analysed here were photographed towards the end of the ZIKV outbreak, it is clear that they were not replaced by those containing updated information as the outbreak progressed. A lack of focus on the potential for ZIKV to be sexually or congenitally transmitted this far into the epidemic implies that there may have been a lack of public knowledge about these potential modes of transmission, which is supported by focus group studies conducted at the same time (Bancroft et al., in preparation, [30]), and represents a missed opportunity to significantly reduce the number of ZIKV cases through safe sex practices [24, 31, 32]. It is also possible that difficulty was faced with communicating these risks through posters, particularly due to the stigmatisation of the public discussion of sexual transmission [33, 34].

### A battle against the mosquito

References to an ongoing battle against the mosquito were prevalent amongst the posters, which is consistent with a combative narrative in Brazilian newspapers throughout the ZIKV epidemic, and may be explained by the coincidence of arboviral outbreaks and Brazil’s socio-political instability [35, 36]. Discourse circulated in the media is particularly political during outbreaks [37], so it is important to recognise the possible influence of the ongoing political crisis in 2017 in the messages conveyed

The preventive actions presented across the posters predominantly focused on approaches to control the mosquito, doing so through the promotion of the elimination of breeding sites within the household and the aversion of stagnant water. Community participation in mosquito control through environmental management with water container covers is one of the few approaches shown to be effective against DENV [38], and messages pertaining to the elimination of breeding sites are integral to the prevention of arbovirus transmission [39, 40]. Thus, the focus of these messages in the analysed posters is promising.

The mention of further preventive actions was limited, but in some cases these did extend beyond the control of mosquitoes. Reference to further personal protection measures such as the use of insect repellents or mosquito nets were also limited to one and two posters, respectively. Interviews with pregnant women in the state of Sergipe, Brazil during the ZIKV outbreak found that 100/177 participants (56%) reported using repellents, and that a lack of advice, as well as lower economic and social conditions, had a negative effect on their usage [41]. Although the value of repellents and bed nets in the prevention of arbovirus transmission has not been fully established [38], these tools are considered among the most important available for personal protection [42–44].

Finally, family planning services were not featured in any of the posters analysed, but were identified by Pinchoff, et al. [40] as among the evidence-based preventive behaviours with a high potential to strengthen social and behaviour change in the response to ZIKV. Other behaviours recommended in this particular study included the use of repellents, the use of condoms during pregnancy, and seeking antenatal care. The general absence of these recommendations across the posters may be related to cultural or religious factors, and implies that, like the range of modes of transmission, the breadth of preventive actions promoted was insufficient to effectively reduce the transmission of ZIKV, in particular.

### A responsibility to protect and prevent

The sense of personal responsibility conveyed by the posters focused not only on the personal protection of the reader but held individuals accountable for the protection of those around them. One poster emphasised the need to protect pregnant women and their babies, but failed to promote preventive actions relating to the sexual or congenital transmission of ZIKV. A failure to incorporate these messages into the posters to a greater extent highlights a missed opportunity to protect a highly vulnerable population. Correspondingly, only one poster addressed the importance of the use of contraceptives, and the message was brief, simply advising readers to use a condom. The government’s dissemination of this message assumes that women across Brazil possess a degree of reproductive autonomy. This assumption has the potential to influence vulnerability to ZIKV, and thus the ability to engage in preventive behaviours, and affects the capacity of these messages to induce behaviour change.

Whilst there was limited emphasis on the need to specifically exert protection over pregnant women and their babies, who were much more vulnerable to adverse outcomes following ZIKV infection [45], many of the posters also referenced the importance of collective action. This suggests a degree of a shared responsibility to prevent arboviral transmission. The framing of these messages as a shared responsibility that addressed both individual and collective action echoes the findings of Ribeiro, et al. [35]. The messages held individuals and communities accountable for the spread of the arboviruses, resting a burden on them as opposed to placing a responsibility on the state or addressing the wider determinants of disease transmission, such as poverty and public service provision [46–48].

### The health belief model

The HBM has been successfully applied in diverse cultural and topical contexts. The model posits that individuals are influenced to engage in behaviours that protect their health through six belief variables [15]. A noted limitation of the HBM is that it fails to specify variable ordering, whereby some variables may mediate relationships in parallel, in sequence, or in tandem with a moderator [49]. For example, exposure to a poster campaign could increase self-efficacy, which in turn could influence perceived barriers, and these could predict behaviour. Nonetheless, it has been identified that, of the six variables, perceived barriers and perceived benefits are the strongest predictors of behaviour [50], and should be targeted in communication campaigns to promote health. The content analysis revealed that perceived benefits was the least commonly identified variable amongst the posters analysed. The underutilisation of this variable, that preventive actions would benefit the individual [51], suggests that the posters may have not fully engaged individuals in positive health behaviours. Indeed, Janz and Becker [16] argue that an individual’s perceived benefits need to outweigh the perceived barriers to induce behaviour change.

The messages on the analysed posters addressed a variety of potential barriers, and tackled the perceived complexity of preventive actions by providing alternative actions and visual explanations of how to eliminate breeding sites. However, messages utilising this belief variable were still only present across 43% of the posters. An individual may perceive themselves to be susceptible to the arbovirus, and they may also agree that engaging in preventive actions could be effective for reducing this susceptibility, but without reducing the perceived barriers to engaging in these actions, there may be limited scope for behaviour change [15, 16].

Messages targeting perceived severity and perceived susceptibility were identified to a similar extent across the posters. Although our understanding of the subsequent development of congenital syndromes and, hence, the particular threat to pregnant women and their children, evolved throughout the epidemic, a lack of warning about CZS in 2017 risks a lack of acknowledgement of the severity of the virus and its outcomes in the population.

There are a number of limitations to this study. Whilst the HBM can be used to explore the potential effectiveness of posters in inducing behaviour change and, thus, preventing the transmission of arboviruses, it is important to acknowledge that we cannot fully evaluate their importance because they were used alongside other modes of communication and indeed other efforts to prevent ZIKV, DENV and other arbovirus transmission in Brazil in 2017 [52]. There are many potential biases and unknown factors that would make it difficult to evaluate the impact of the posters and their messages.

## Conclusions

This study has revealed that posters presented to the Brazilian public did not fully capture the range of disease transmission modes or the range of methods available to reduce risk, particularly to pregnant women, and we encourage future campaigns to address these limitations. In addition, the development of future risk communication posters should carefully consider the incorporation of components that highlight the potential benefits to engaging in preventive behaviours and means for tackling the potential barriers that may arise within a target population. Indeed, this is consistent with recommendations made following an evaluation of Instagram posts about ZIKV for public health communication [53]. Moreover, when encouraging individuals to engage in preventive behaviours through the use of cues to action, appropriate instructions should be paired with both slogans and commands. This is necessary to ensure that individuals obtain the information that they require to prevent arboviral transmission. Coupled with this is the need to replace outdated posters as knowledge about an outbreak develops, to ensure that individuals are aware of additional risks.

In order to develop suitable public health messages and strengthen arbovirus risk communication, further efforts are also required to identify the determinants of arbovirus infection and barriers to engaging in preventive actions. This should include an investigation of the perceptions of arboviruses amongst the population [54]. Finally, further studies are required to strengthen existing evidence for the value of vector-control interventions to facilitate the appropriate development of messages to prevent arbovirus transmission by mosquitoes and thus, minimise the incidence of arboviral disease [38].

## Supplementary Information

**Additional file 1.**

**Additional file 2.**

## Data Availability

All data generated or analysed during this study are included in this published article. We have photographed the posters, but we donot own the copyright of the posters and they should not be inlcuded in the article's Creative Commons licence.

## Abbreviations

CHIKV: Chikungunya Virus
CZS: Congenital Zika Syndrome
DENV: Dengue Virus
HBM: Health Belief Model
YFV: Yellow Fever Virus
ZIKV: Zika Virus
