# Supplementary material for: Public health messages on arboviruses transmitted by Aedes aegypti in Brazil
Source: BMC Public Health. 2021 Jul 9;21:1362. doi: 10.1186/s12889-021-11339-x (PMC8272386; doi:10.1186/s12889-021-11339-x)
Supplement: Supplementary file 1 — Additional file 1. [file 12889_2021_11339_MOESM1_ESM.docx]

**Public Health Messages on Arboviruses Transmitted by *Aedes aegypti* in Brazil**

India L. Clancy^1†^, Robert T. Jones^2†^, Grace M. Power^2,3^, James G. Logan^2*^, Jorge Alberto Bernstein Iriart^4^, Eduardo Massad^5^, John Kinsman^6^.

^1^Department of Public Health, Environments & Society, London School of Hygiene & Tropical Medicine, London, United Kingdom; ^2^Department of Disease Control, London School of Hygiene & Tropical Medicine, London, United Kingdom; ^3^ MRC Integrative Epidemiology Unit, Population Health Sciences, Bristol Medical School, University of Bristol, United Kingdom; ^4^Institute of Collective Health, Universidade Federal da Bahia, Salvador, Brazil.

^5^School of Applied Mathematics, Fundacao Getulio Vargas, Rua Praia de Botafogo 190, Rio de Janeiro, CEP 22250-900, RJ, Brazil; ^6^Department of Epidemiology and Global Health, Faculty of Medicine, Umeå University, Umeå, Sweden

*corresponding author

^†^Contributed equally to this work

**Supplementary Material 1.**

**Full set of posters**

**
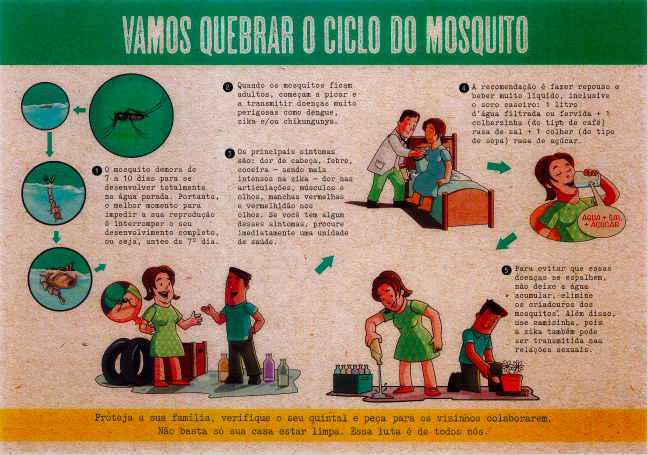
**

**National Poster 1 (N1)**

**
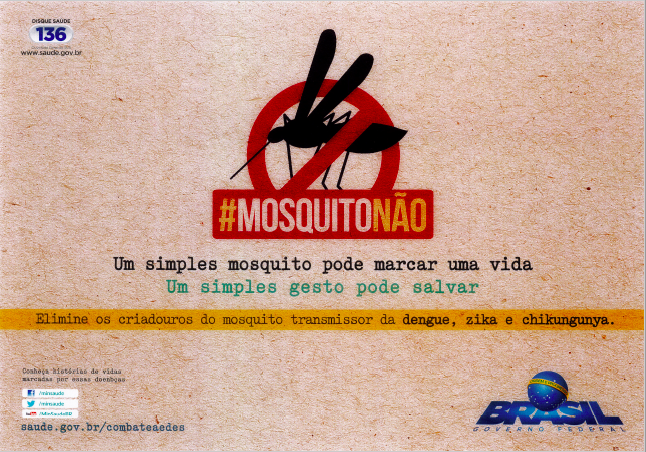
**

**National Poster 2 (N2)**

**
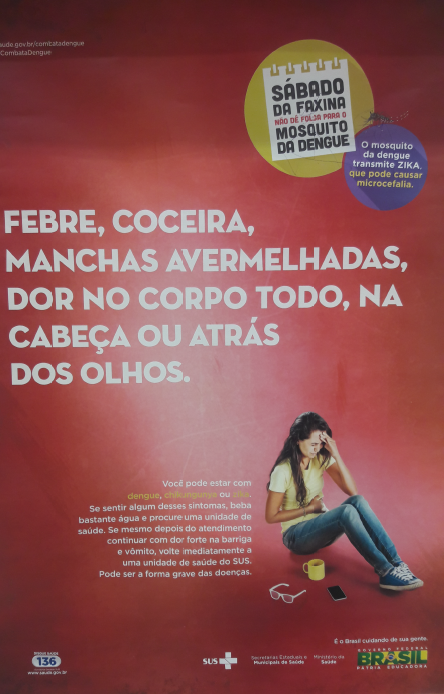
**

**National Poster 3 (N3)**

**
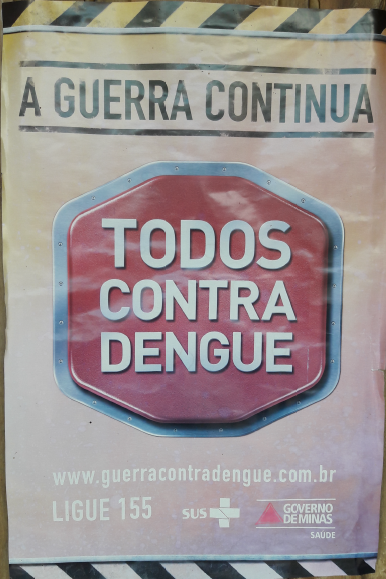
**

**National Poster 4 (N4)**

**
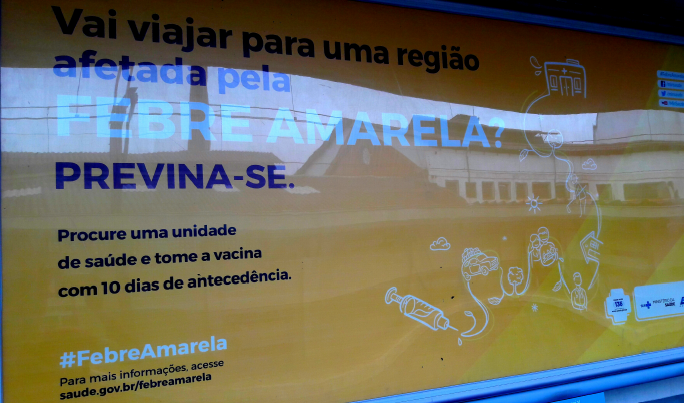
**

**National Poster 5 (N5)**

**
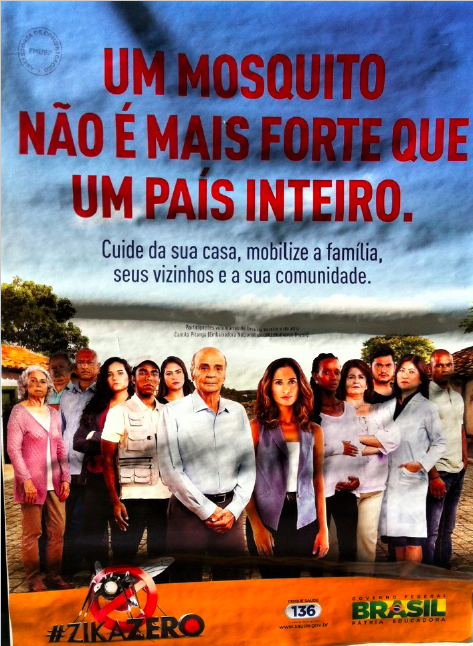
**

**National Poster 6 (N6)**

**
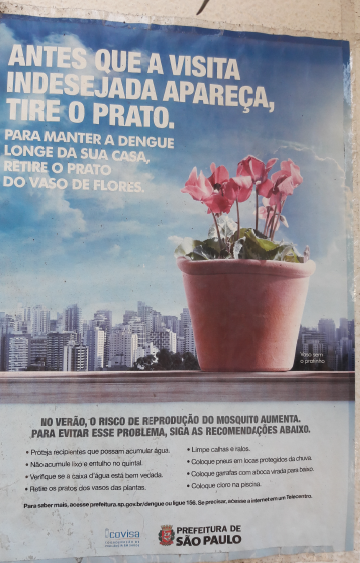
**

***São Paulo Poster 1 (SP1)***

***
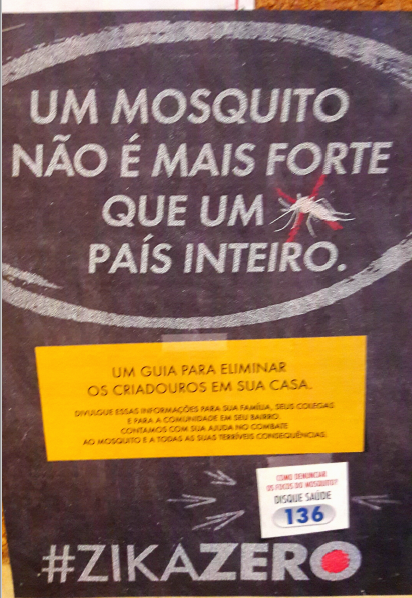
***

**
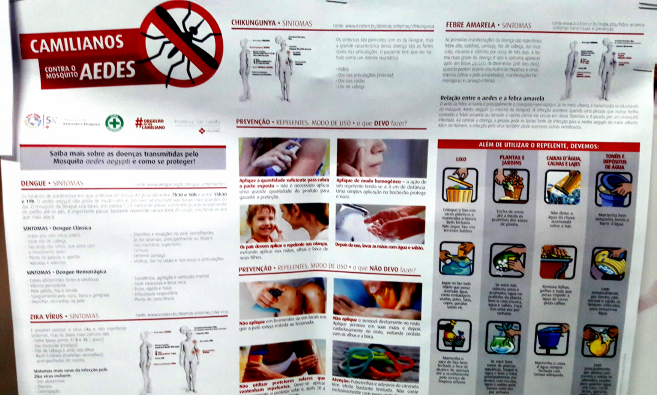
**

***São Paulo Poster 2 – Pages 1 & 2 (SP2)***

***
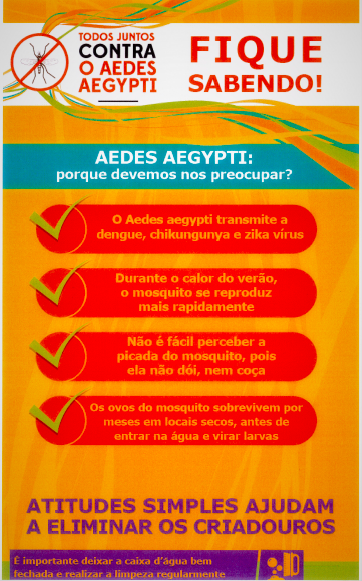
***

***São Paulo Poster 3 (SP3)***

***
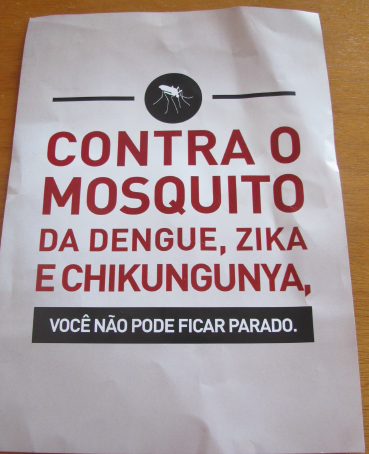
***

***São Paulo Poster 4 (SP4)***

***
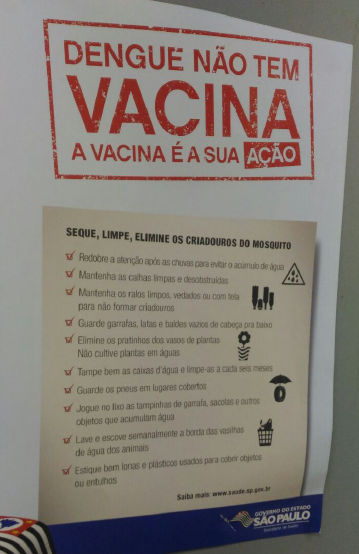
***

***São Paulo Poster 5 (SP5)***

***
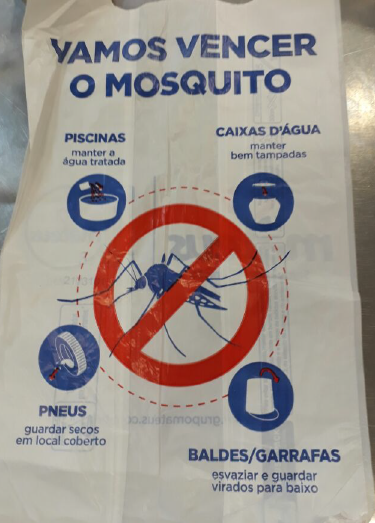
***

***São Paulo Poster 6 (SP6)***

***
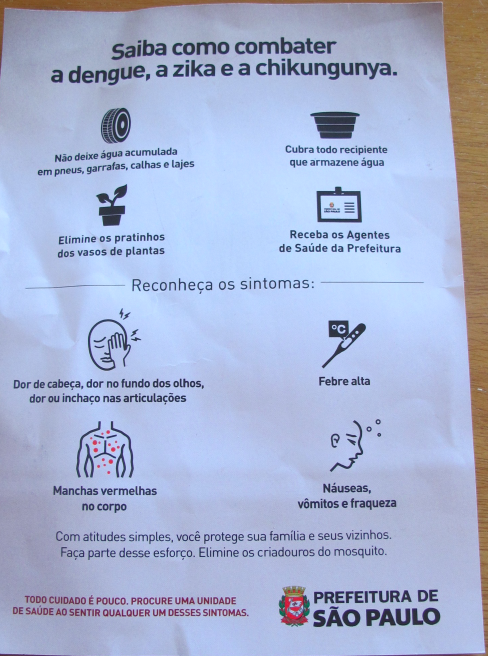
***

***São Paulo Poster 7 (SP7)***


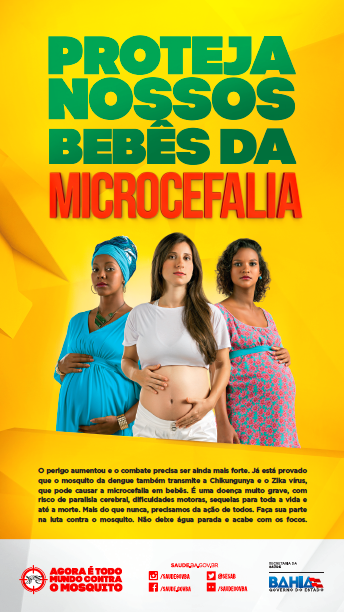


***Salvador Poster 1 (S1)***


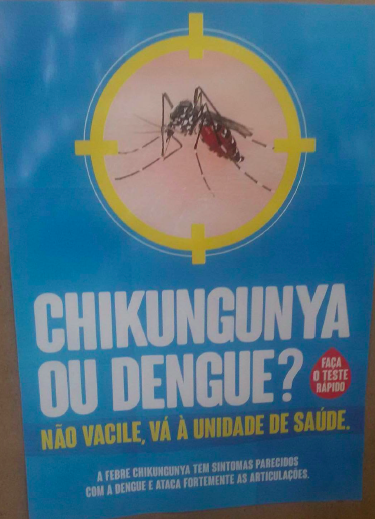


***Salvador Poster 2 (S2)***


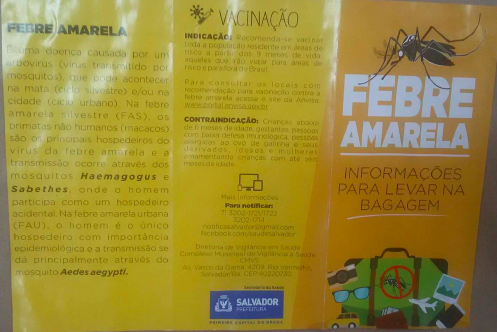

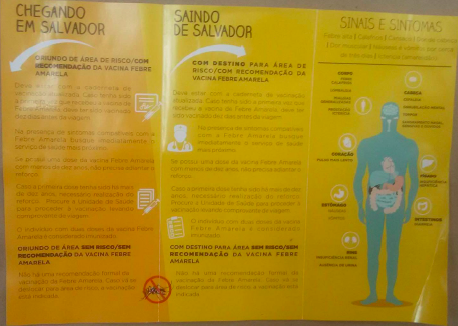
***Salvador Poster 3 – Pages 1 & 2(S3)***

***
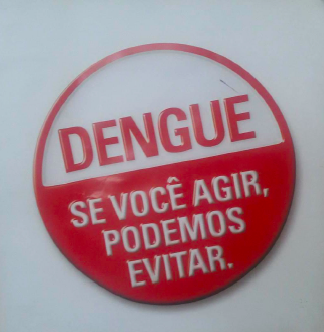
***

***Salvador Poster 4 (S4)***

***
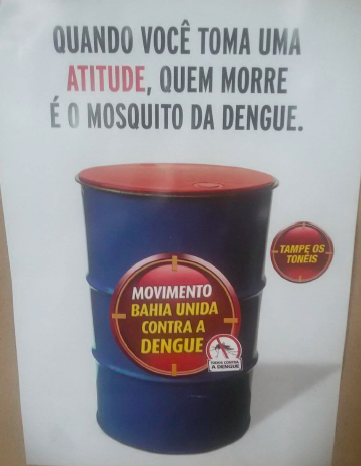
***

***Salvador Poster 5 (S5)***

***
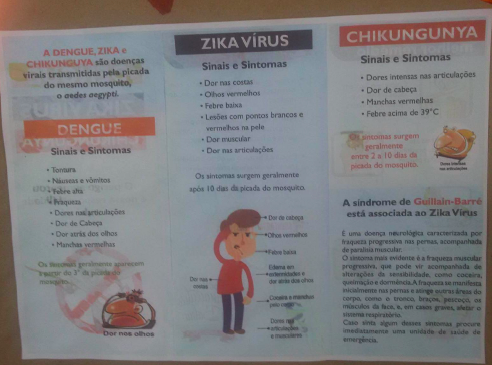
***

***Salvador Poster 6 (S6)***

***
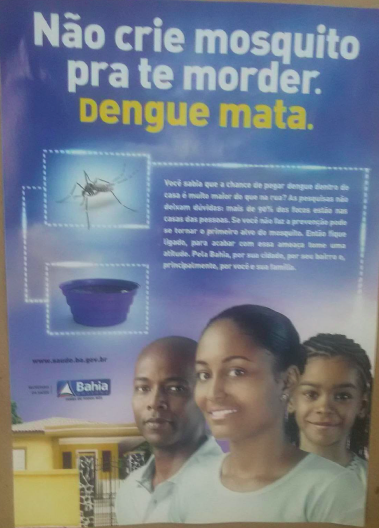
***

***Salvador Poster 7 (S7)***

***
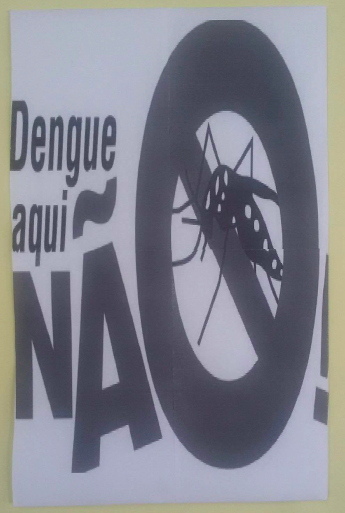
***

***Salvador Poster 8 (S8)***

***
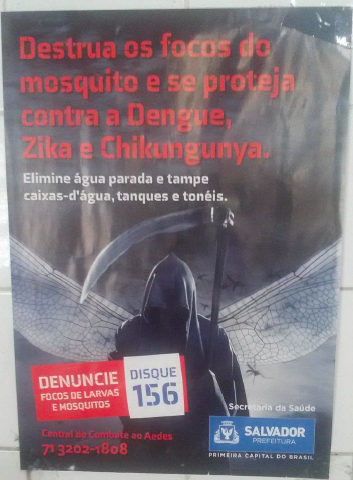
***

***Salvador Poster 9 (S9)***

***
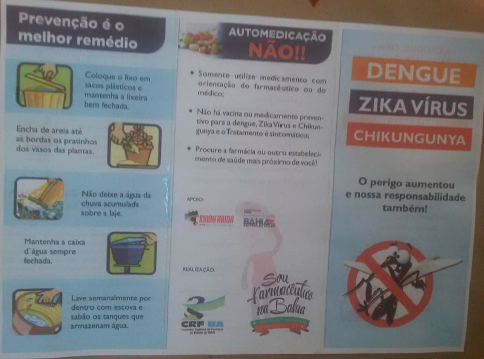
***

***Salvador Poster 10 (S10)***

***
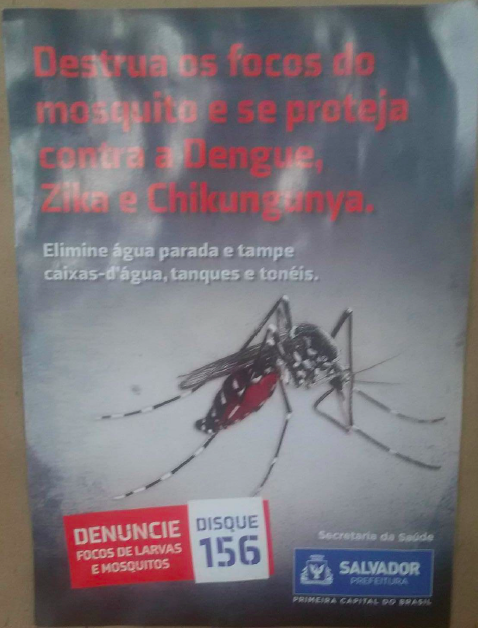
***

***Salvador Poster 11 (S11)***

***
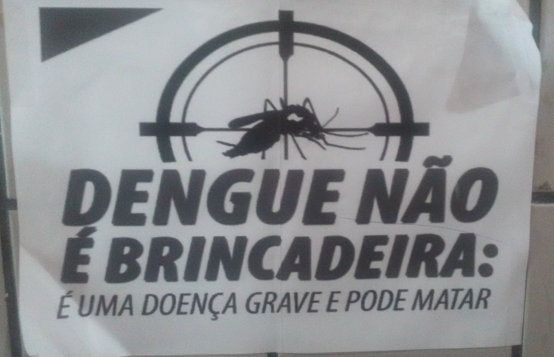
***

***Salvador Poster 12 (S12)***

***
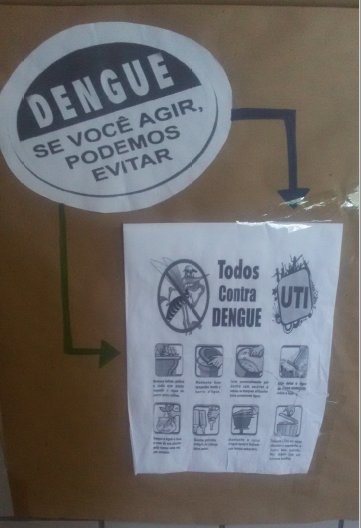
***

***Salvador Poster 13 (S13)***

***
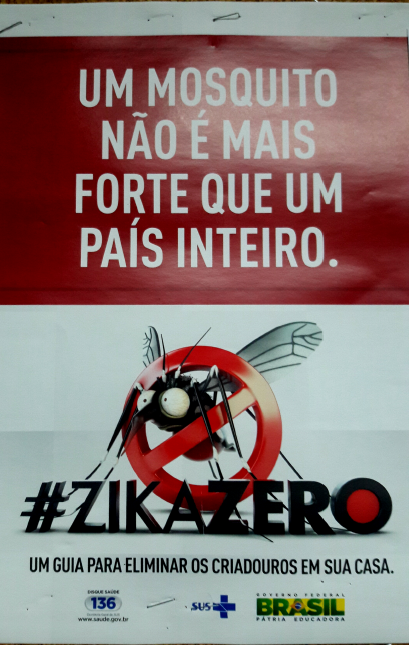
***

***
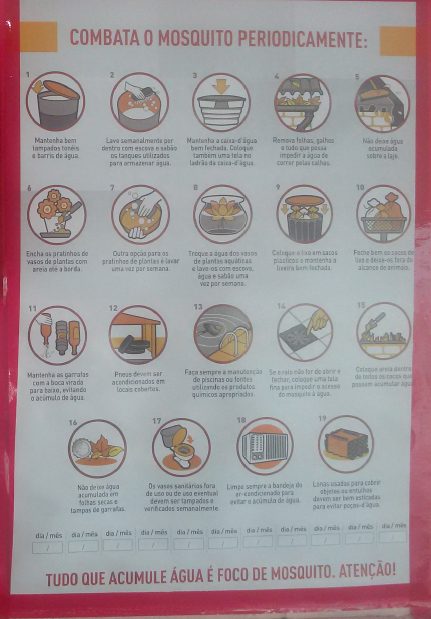
***

***Salvador Poster 14 – Pages 1 & 2 (S14)***

***
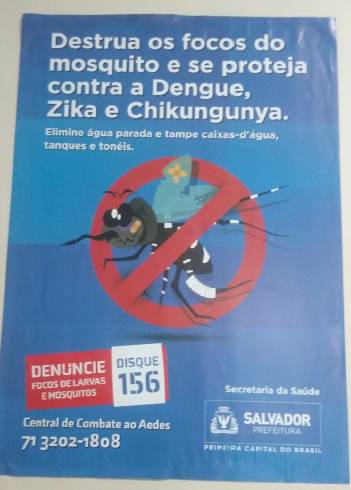
***

***Salvador Poster 15(S15)***

***
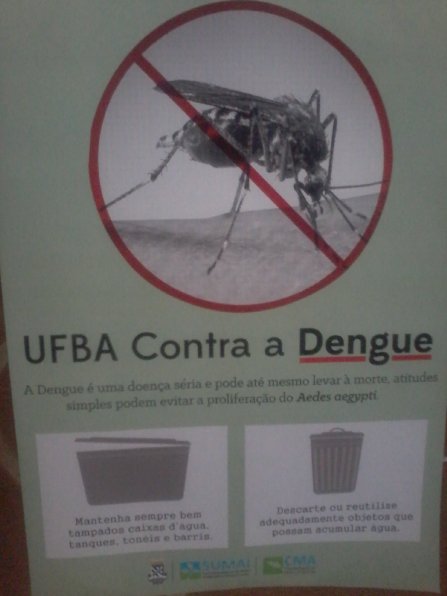
***

***Salvador Poster 16 (S16)***

***
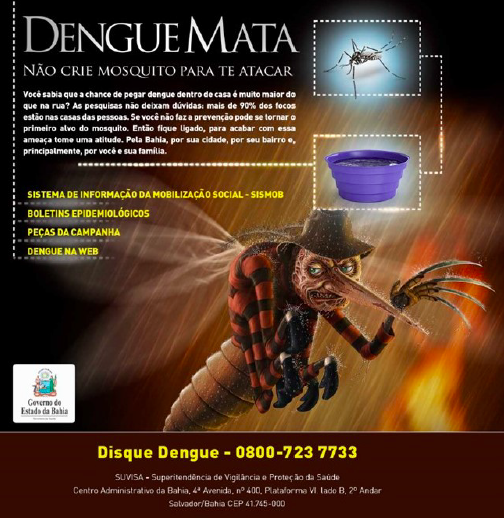
***

***Salvador Poster 17 (S17)***

***
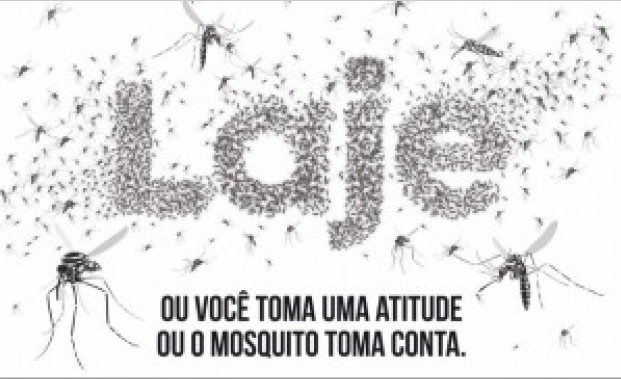
***

***Salvador Poster 18 (S18)***

***
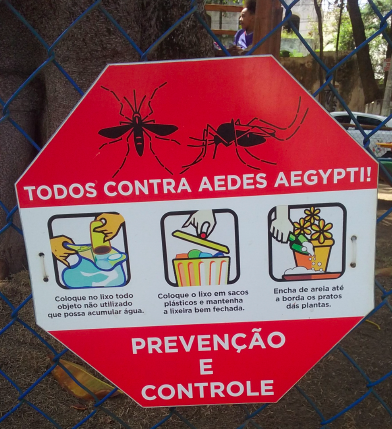
***

***Salvador Poster 19 (S19)***

***
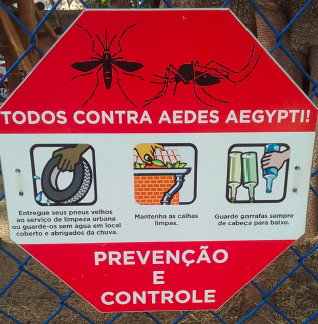
***

***Salvador Poster 20 (S20)***

***
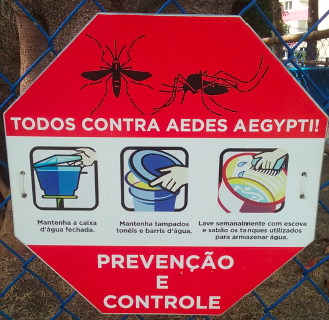
***

***Salvador Poster 21 (S21)***


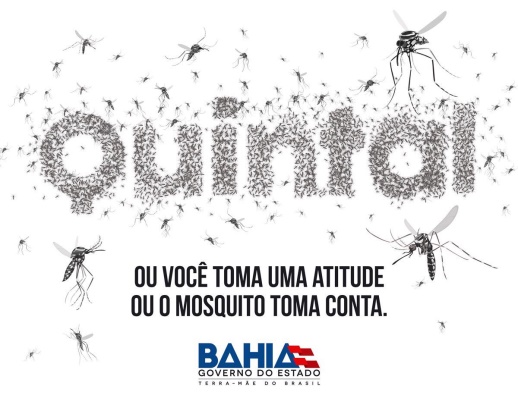


***Salvador Poster 22 (S22)***


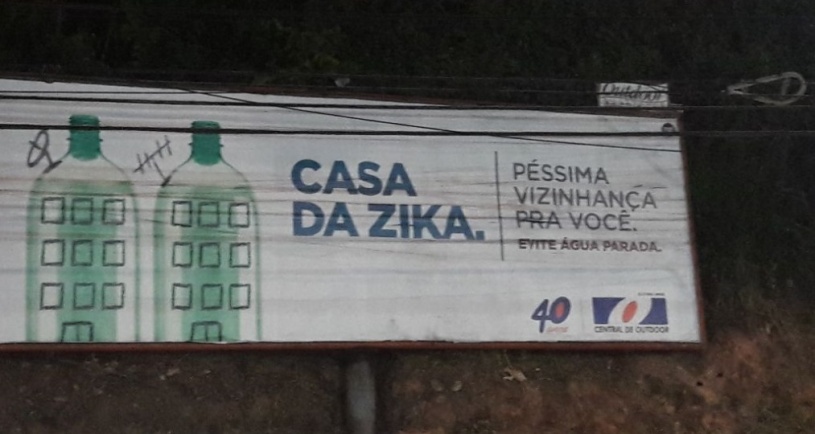


***Salvador Poster 23 (S23)***


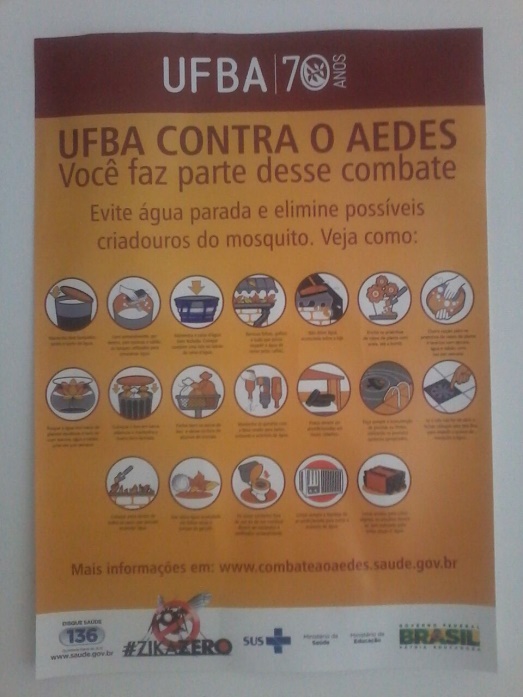


***Salvador Poster 24 (S24)***
