# Supplementary material for: Public health messages on arboviruses transmitted by Aedes aegypti in Brazil
Source: BMC Public Health. 2021 Jul 9;21:1362. doi: 10.1186/s12889-021-11339-x (PMC8272386; doi:10.1186/s12889-021-11339-x)
Supplement: Supplementary file 2 — Additional file 2. [file 12889_2021_11339_MOESM2_ESM.docx]

**Public Health Messages on Arboviruses Transmitted by *Aedes aegypti* in Brazil**

India L. Clancy^1†^, Robert T. Jones^2†^, Grace M. Power^2,3^, James G. Logan^2*^, Jorge Alberto Bernstein Iriart^4^, Eduardo Massad^5^, John Kinsman^6^.

^1^Department of Public Health, Environments & Society, London School of Hygiene & Tropical Medicine, London, United Kingdom; ^2^Department of Disease Control, London School of Hygiene & Tropical Medicine, London, United Kingdom; ^3^ MRC Integrative Epidemiology Unit, Population Health Sciences, Bristol Medical School, University of Bristol, United Kingdom; ^4^Institute of Collective Health, Universidade Federal da Bahia, Salvador, Brazil.

^5^School of Applied Mathematics, Fundacao Getulio Vargas, Rua Praia de Botafogo 190, Rio de Janeiro, CEP 22250-900, RJ, Brazil; ^6^Department of Epidemiology and Global Health, Faculty of Medicine, Umeå University, Umeå, Sweden

*corresponding author

^†^Contributed equally to this work

**Supplementary Material 2**

**Poster translations**

**National Poster 1 (N1)**

Let's break the mosquito cycle.

1. The mosquito takes 7 to 10 days to fully develop in standing water. Therefore, the best time to prevent its reproduction is to stop its full development, that is, before the 7th day.

2. When mosquitoes become adults, they begin to sting and transmit very dangerous diseases such as dengue, zika and / or chikungunya.

3. The first symptoms are: headache, fever, itching - being more intense in the zika - pain in the joints, muscles and eyes, red spots and redness in the eyes. If you have any of these symptoms, seek immediate medical attention.

4. The recommendation is to rest and drink plenty of fluids, including homemade serum (or Oral Rehydration Therapy – ORT): 1 liter of filtered or boiled water + 1 teaspoon of shallow salt + 1 tablespoon of sugar.

5. To prevent these diseases from spreading, do not let water accumulate, eliminate mosquito breeding grounds. Also, use a condom, as the zika can also be transmitted during sex.

Protect your family, check your yard and ask the neighbors to cooperate. It is not enough that your house is clean. This fight belongs to all of us.

**National Poster 2 (N2)**

#MOSQUITONO

A simple mosquito can make a living (or can leave a mark on a life)

A simple gesture can save

Eliminate the breeding sites of mosquitoes transmitting dengue, zika and chikungunya

Know life stories marked by these diseases

**National Poster 3 (N3)**

Saturday cleaning. Do not give the dengue mosquito any gap (or opportunity).

The dengue mosquito transmits zika, which can cause microcephaly.

Fever, itching, red spots, pain in the whole body, in the head or behind the eyes.

You may be with dengue, chikungunya or zika. If you experience any of these symptoms, drink plenty of water and seek a health center. If even after the care you continue with severe pain in the belly and vomit, immediately return to a SUS health unit. It can be the severe form of disease.

**National Poster 4 (N4)**

The war continues

All against dengue fever

www.guerracontradengue.com.br

Call 555

**National Poster 5 (N5)**

Are you going to travel to a region affected by yellow fever?

Prevent yourself.

Look for a health facility and take the vaccine 10 days in advance.

#Yellow Fever

For more information, go to saude.gov.br/febreamarela

**National Poster 6 (N6)**

A mosquito is not stronger than an entire country.

Take care of your home, mobilize your family, your neighbors and your community

**São Paulo Poster 1 (SP1)**

Before the unwanted visit appears, remove the dish.

To keep dengue away from your home, remove the dish from the flower pot.

In summer, the risk of mosquito breeding increases.

To avoid this problem, follow the recommendations below.

• Protect containers that may collect water.

• Do not accumulate litter and debris in the yard.

• Check that the water tank is well sealed.

• Remove dishes from plant pots.

• Clean gutters and drains.

• Place tires in places protected from rain.

• Place bottles with their mouths down.

• Put chlorine in the pool.

To find out more, go to prefeitura.sp.gov.br/dengue or call 156. If it is necessaire, access the Internet at a Telecentre.

Photo caption: Vase without the dish

**São Paulo Poster 2 (SP2)**

A mosquito is not stronger than an entire country.

A guide to eliminate breeding sites in your home.

Share this information to your family, your colleagues, and the community in your neighborhood. We count on your help in combating the mosquito and all its terrible consequences.

How to report mosquito outbreaks?

Dial Health 136

#zikazero

CAMILIANOS AGAINST MOSQUITO AEDES

Learn more about Mosquito Aedes aegypti and how to protect yourself

DENGUE - SYMPTOMS

The hours of predominance in which the female of the mosquito bites are between 7:30 a.m. and 10:00 a.m. and between 3:30 a.m. and 7:00 p.m. Aedes aegypti does not like much heat and so it hides in the hottest hours of the day. The dengue mosquito flies low, averaging 1.20 in height. As it bites mainly from the knee to the feet, it is important to get quite repellent in this area of ​​the body at the times it attacks the most.

SYMPTOMS - DENGUE CLASSIC

• High fever with sudden onset

• Splitting headache

• Pain behind the eyes, which worsens with their closing

• Loss of taste and appetite

• Nausea and vomiting

• Measles-like spots and skin eruptions, especially in the chest and upper limbs

• Extreme tiredness

• Clogging, pain in the body and bones and joints

SYMPTOMS - DENGUE HEMORRHAGIC

• Strong and continuous abdominal pain

• Persistent vomiting

• Pale, cold, moist skin

• Bleeding from nose, mouth and gums

• Red spots on the skin

• Drowsiness, restlessness and mental confusion

• Excessive thirst and dry mouth

• Difficulty breathing

• Loss of consciousness

Zika Virus Symptoms

It is possible to contract the Zika virus and not manifest symptoms, but the most common signs are:

• Low fever (between 37.8 and 38.5 degrees)

• Muscle pain (myalgia)

• Headache and behind the eyes

• Skin rash (red spots, accompanied by itching)

More rare symptoms of Zika virus infection include:

• Abdominal pain

• Diarrhea

• Cold

• Photophobia

• Small ulcers in the oral mucosa.

CHIKUNGUNYA - SYMPTOMS

The symptoms are similar to those of dengue, but the great characteristic of this disease are the severe pains in the joints. The patient has to be treated as a rheumatic patient.

• Fever

• Joint pain (severe)

• Back pain

• Headache

YELLOW FEVER - SYMPTOMS

The first manifestations of the disease are sudden: high fever, chills, tiredness, headache, muscle pain, nausea and vomiting for about three days. The most serious form of the disease is rare and usually appears after a brief period of well-being (up to two days), when hepatic and renal insufficiencies, jaundice (yellow eyes and skin), hemorrhagic manifestations and intense tiredness may occur.

Relationship between Aedes and yellow fever

The vector of yellow fever is mainly the mosquito Haemagogus. In the urban area, the transmission occurs through the mosquito Aedes aegypti (the same as dengue). The infection happens when a person who has never contracted yellow fever or taken the vaccine against it circulates in forest areas and is bitten by an infected mosquito. By contracting the disease, a person can become a source of infection for Aedes aegypti in urban areas. In addition to the man, the virus infection can also affect other vertebrates.

PREVENTION - REPELLENTS - METHOD OF USE - what should I do?

Apply sufficient amount to cover the exposed part - it is not necessary to apply a large amount to ensure protection.

Apply homogeneously - the action of a repellent is limited to 4 cm away. A simple application on the cheek protects the nose.

Parents should apply the repellent on children, avoiding applying on the hands, eyes and mouth of their children.

After use, wash hands with soap and water

PREVENTION - REPELLENT - METHOD OF USE - what should NOT I do?

Do not apply on wounds or where skin is irritated or injured.

Do not apply aerosol directly on face. Apply first on your hands and then carefully on the face, avoiding eye and mouth contact.

Do not use sunscreens that contain repellents. First apply the sunscreen and after 20 to 30 minutes apply the repellent.

Attention: Citronella bracelets and stickers have a very limited effect. Do not rely exclusively on these methods.

IN ADDITION TO USING THE REPELLENT, WE MUST:

TRASH

Place trash in plastic bags and keep trash tightly closed. Do not throw garbade in abandoned land.

Throw away any objects that may accumulate water such as packaging, pots, cans, bottles, vases, etc.

Keep the garbage bag tightly closed and out of reach of the animals until collected by the urban cleaning service.

PLANTS AND GARDENS

Fill the bowls of the plant pots with sand to the brim.

If you did not put sand and accumulated water in the dish of the plant, wash it with brush, water and soap. Do this once a week.

If you have pots of aquatic plants, change the water and wash the pot mostly from the inside with brush, water and soap at least once a week.

WATER BOXES, SHELVES AND LAJES

Do not let rainwater accumulate on the slab.

Remove leaves, twigs, and anything else that may prevent water from flowing through the gutters.

Keep the water box always closed, with a suitable lid.

TONES AND WATER TANKS

 Keep well covered casks and barrels of water.

Wash the tanks used to store water, especially with soap and water.

Wash the reservoirs to store water at home, such as jugs, bottles, jars, etc. with soap and brush.

**São Paulo Poster 3 (SP3)**

ALL TOGETHER AGAINST THE AEDES AEGYPTI

KNOW IT!

AEDES AEGYPTI - WHY SHOULD WE WORRY?

• Aedes aegpyti transmits dengue, chikungunya and zika virus

• During the summer heat, the mosquito breeds faster

• It is not easy to see the mosquito bite, because it does not hurt or scratch

• Mosquito eggs survive for months in dry places before entering the water and turning into larvae.

SIMPLE ATTITUDES HELP ELIMINATE CREATORS

It is important to keep the water box tightly closed and to clean it regularly.

**São Paulo Poster 4 (SP4)**

Against the dengue, zika and chikungunya mosquito, you can not stand still

**São Paulo Poster 5 (SP5)**

Dengue has no vaccine

The vaccine is your action

Dry, clean, eliminate mosquito breeding sites

• Reduce attention after rainfall to eliminate water accumulation

• Keep gutters clean and unobstructed

• Keep the drains clean, sealed, or screened so as not to form a breeding ground.

• Store empty bottles, cans and buckets upside down

• Remove pots from plant pots. Do not grow plants in

• Cover water wells and clean them every six months

• Store tires indoors

• Throw away the bottle caps, bags and other objects that accumulate water

• Weekly wash and brush the edges of animal water containers

• Stretch tarps and plastics used to cover objects or debris

**São Paulo Poster 6 (SP6)**

LET'S WIN THE MOSQUITO

POOLS

Maintain the water treated

WATER BOXES

Keep them well capped

TIRES

Keep them dry in an open place

BUCKETS / BOTTLES

Empty and store them with the face down

**São Paulo Poster 7 (SP7)**

Learn how to combat dengue, zika and chikungunya.

Do not leave water accumulated on tires, bottles

Cover any container that collects water

Remove the dishes from the potted plants

Get Health Agents from City Halls

Acknowledge the symptoms:

Headache, pain in the back of the eyes, pain or swelling in the joints

High fever

Red spots on the body

Nausea, vomiting and weakness

With simple attitudes, you protect your family and your neighbors.

Be part of that effort. Eliminate mosquito breeding sites.

YOU CAN’T BE TOO CAREFUL. SEEK A HEALTH UNIT WHEN YOU FEEL ANY OF THESE SYMPTOMS.

**Salvador Poster 1 (S1)**

Protect our babies from microcephaly

Risk has risen and the battle needs to be even tougher. It´s already proven than a dengue mosquito also transmits Chikungunya and Zika virus, which can cause microcephaly amongst babies. It´s a very serious illness, with risk of cerebral palsy, motor difficulties, lifelong sequels and even death. Everyone needs to take action now more than ever. Do your part in this fight against the mosquito. Don´t leave stagnant water and eliminate its breeding sites.

Logo with mosquito picture: Now it´s everyone against the mosquito

**Salvador Poster 2 (S2)**

Chikungunya or dengue? Do the rapid test.

Don´t hesitate, go to a health unit.

Chikungunya fever has similar symptoms to dengue and it severely affects joints.

**Salvador Poster 3 (S3)**

YELLOW FEVER

It´s an illness caused by an arbovirus (virus which is transmitted by mosquitoes), which can take place in the wild (and or in the city (urban cycle). In wilderness yellow fever, non-human primates (monkeys) are the main hosts for the yellow fever virus and transmission happens through two mosquitoes, Haemagogus and Sabethes, with human beings participating as accidental hosts.

In regards to urban yellow fever (???), human beings are the only hosts with epidemiologic importance and transmission usually occurs through the Aedes aegypti mosquito.

VACCINATION

INDICATION: It´s recommended to vaccine the whole population ages 9 months and older who reside in areas of risk, and people who will travel to areas of risk or outside Brazil.

If you need to find out which are the locations with recommendation of vaccination against yellow fever, check the Anvisa site: www.portal. anvisa.gov.br

CONTRAINDICATIONS:

Children under the age of 6 months, pregnant women, people with low immune defense, allergic to chicken egg and its derivatives, elderly and women who breast feed children of up to 6 months of age.

More information: links, phones, etc.

YELLOW FEVER

INFORMATION TO CARRY ON YOUR LUGGAGE

Arriving in Salvador

If you´re from an area of risk or with recommendation of yellow fever vaccination

You must have your vaccination card up to date. If it´s the first time you received the yellow fever vaccine, you must have been vaccinated ten days before your trip.

In case you have any potential symptoms of yellow fever, immediately go to the closest health unit.

If you were vaccinated with one dose of yellow fever vaccine less than ten years ago, you don´t need to speed up to get the booster shot.

In case you received your first dose more than ten years ago, you need the booster shot. You need to go to a health unit to proceed to receive your vaccination and bring your travel ticket.

People who have received two doses of yellow fever vaccination are considered to be immunized.

IF YOU´RE FROM AND AREA WITHOUT RISK OR RECOMMENDATION OF YELLOW FEVER VACCINATION

There is no formal recommendation on yellow fever vaccination. Vaccination is indicated if you´re traveling to an area of risk.

Traveling away from Salvador

Towards an area of risk or with recommendation of yellow fever vaccination

You must have your vaccination card up to date. In case it´s the first time you receive a yellow fever vaccine, you must have been vaccinated ten days before your trip.

In case of yellow fever-likesymptoms you have to go immediately to the closest health unit.

If you were vaccinated with one dose of yellow fever vaccine less than ten years ago, you don´t need to speed up to get the booster shot.

In case you received your first dose more than ten years ago, you need the booster shot. You need to go to the health services unit to proceed to receive your vaccination and bring your travel ticket.

People who have received two doses of yellow fever vaccination are considered to be immunized.

If you´re traveling to an area without risk or recommendation of yellow fever vaccination

There is no formal recommendation on yellow fever vaccination. Vaccination is indicated if you´re traveling to an area of risk.

Signs and symptoms:

High temperature _ shivering _ tiredness _ headache _ muscle pain _ nausea and vomit episodes for about three days _ jaundice

Body

Fever

Shivering

Low back pain

Generalized myalgia

Prostration

Jaundice

Heart

Lower heart rate

Stomach

Nausea

Vomit

Kidneys

Renal insufficiency

Absence of urine

Head

Headache

Mental obnubilation

Numbness

Nose, gums and ear bleeding

Liver

Liver insufficiency

Intestines

Diarrhea

**Salvador Poster 4 (S4)**

DENGUE

IF YOU TAKE ACTION, WE CAN AVOID IT.

**Salvador Poster 5 (S5)**

WHEN YOU TAKE RESPONSIBILITY, THE ONE THAT DIES IS THE DENGUE MOSQUITO.

Put lids on buckets.

“Bahia united against dengue” Movement

Everyone against dengue

**Salvador Poster 6 (S6)**

DENGUE, ZIKA and CHIKUNGUNYA are viral illnesses which are transmitted by bites of the same mosquito, aedes aegypti.

DENGUE

Signs and symptoms

Dizziness

Nausea and vomit

High fever

Weakness

Aching joints

Headache

Pain behind the eyes

Red stains

Symptoms normally appear after the third day of the mosquito bite.

Picture: aching eyes

ZIKA VIRUS

Signs and symptoms

Back ache

Red eyes

Low fever

Lesions with white and red dots in the skin

Muscular pain

Aching joints

Symptoms generally appear after ten days of the mosquito bite.

Headache, red eyes, low fever, back pain, edema in extremities and pain behind the eyes, itchiness and stains in the body, muscle and joints pain.

CHIKUNGUNYA

Signs and symptoms

Intense joints pain

Headache

Red stains

Fever above 39°C

Symptoms generally appear between 2 and 10 days after the mosquito bite.

PICTURE: intense joints pain

Guillain-Barré syndrome is associated to Zika virus

It´s a neurological illness which is characterized by progressive weakness in legs, along with muscle paralysis.

The most evident symptom is progressive muscular weakness, which can come along with sensitive alterations, such as itchiness, blazing and numbness. Weakness affects mainly legs and affects other body parts, such as the upper body, arms, neck, facial muscles and, in very serious cases, the respiratory system.

In case you feel any of those symptoms, immediately seek for an emergency health unit.

**Salvador Poster 7 (S7)**

Don´t breed the mosquito for it to bite you. Dengue kills.

Did you know that the chance of getting sick with dengue inside your house is much higher than in the street? Studies leave no doubt: more than 90% of the focuses are in people’s houses. If you don´t prevent it, you can turn out to be the first target of the mosquito.

Pay attention, in order to finish up with this threat, take responsibility. For Bahia, for your city, for your neighbourhood and mainly for you and your family.

www. saude.ba.gov.br

**Salvador Poster 8 (S8)**

DENGUE… NOT HERE!

**Salvador Poster 9 (S9)**

Destroy the mosquito breeding sites and protect yourself against Dengue, Zika and Chikungunya.

Eliminate stagnant water, put lids on buckets, tanks and barrels.

Report larvae and mosquito and breeding points. Dial 156.

**Salvador Poster 10 (S10)**

Prevention is the best remedy

Place your garbage in plastic bags and keep the garbage can properly closed.

Fill flower pots with sand up to the edge.

Don´t let rain water accumulate on “laje”

Keep the water tank closed at all times.

Wash buckets where you collect water with a brush and soap weekly.

No self-medication!

Only use medicines with your pharmacist or doctor orientation

There is no vaccine nor preventive medicine for dengue, Zika virus or Chikungunya and treatment is symptomatic.

Look for a pharmacy or your closest health unit!

DENGUE

ZIKA VIRUS

CHIKUNGUNYA

Risk has increased and our responsibility too!

**Salvador Poster 11 (S11)**

Destroy the mosquito breeding sites and protect yourself against Dengue, Zika and Chikungunya.

Eliminate stagnant water, put lids on water tanks, buckets and barrels.

Report larvae and mosquito and breeding points. Dial 156.

**Salvador Poster 12 (S12)**

DENGUE IS NOT A JOKE:

IT´S A SERIOUS ILLNESS AND CAN KILL

**Salvador Poster 13 (S13)**

IF YOU TAKE ACTION, WE CAN AVOID IT

Everyone against DENGUE – UTI

Remove leaves, branches and anything that could prevent water from draining through gutters.

Keep barrels and buckets properly covered.

Wash the buckets you use to store water with a brush and soap on a weekly basis.

Do not let rain water accumulate on the “laje”.

Change the water and wash your flower vases at least once a week.

Always store bottles upside down.

Always keep the water tank closedwith the adequate lid on.

Place your garbage in plastic bags and keep the garbage can properly closed. Don’t throw garbage.

**Salvador Poster 14 (S14)**

A mosquito is not stronger than an entire country.

#Zikazero

A guide to eliminate breeding sites in your home.

PERIODICALLY FIGHT AGAINST THE MOSQUITO

Keep buckets and barrels well covered with lids on.

Wash water storing buckets with brush and soap on a weekly basis.

Keep the water tank properly closed. Also add a mosquito net in the top of water tank (???).

Remove leaves, branches and anything that could possibly prevent water from draining through gutters.

Don’t let water accumulate on the “laje”

Fill plates of flower vases with sand up to the top.

Another option for the plates for plants is to wash them weekly.

Change the aquatic plants water and wash them with a brush, water and soap once a week.

Place the garbage in plastic bags and keep the garbage can properly closed.

Close the garbage bags properly and put them outside and out of reach of animals.

Store bottles upside down, avoiding water from accumulating inside them.

Tires must be fixed in covered locations.

Always maintain swimming pools and water fountains using adequate chemicals.

If your drain is not an open-close one, place a thin net in order to avoid the mosquito from reaching the water.

Place sand in all the broken glass bottles which could accumulate water.

Don’t leave accumulated water in dry leaves and bottle toppers.

Hardly used or non used toilets must be covered and checked on a weekly basis.

Always clean the air conditioning in order to avoid accumulating water.

Canvas used to cover objects or rubble must be tightened in order to avoid water puddles.

Anything that might accumulate water is a mosquito breeding site. Attention!

**Salvador Poster 15 (S15)**

Destroy the mosquito breeding sites and protect yourself against Dengue, Zika and Chikungunya.

Eliminate stagnant water, put lids on water tanks, buckets and barrels.

Report larvae and mosquito and breeding points. Dial 156.

**Salvador Poster 16 (S16)**

UFBA against Dengue

Dengue is a serious illness and can even kill; simple actions can prevent the Aedes aegypti´s proliferation.

Keep water tanks, tanks, buckets and barrels always properly closed with their lids on.

Throw away or adequately reutilize objects that might accumulate water.

**Salvador Poster 17 (S17)**

DENGUE KILLS

Don´t breed the mosquito for it to attack you.

Did you know that the chance of getting sick with dengue inside your house is much higher than in the street? Studies leave no doubt: more than 90% of the bridal sites are in houses. If you don´t prevent it, you can turn out to be the first target of the mosquito. Pay attention (using an informal expression in Portuguese), in order to finish up with this threat, take responsibility. For Bahia, for your city, for your neighbourhood and mainly for you and your family.

INFORMATION SYSTEM OF SOCIAL MOBILIZATION – SISMOB

EPIDEMIOLOGIC BULLETINS

CAMPAIGN PUBLICATIONS

DENGUE IN THE WEB

Dial Dengue: phone numbers

Information

**Salvador Poster 18 (S18)**

Laje

Or you take responsibility or the mosquito takes over.

**Salvador Poster 19 (S19)**

EVERYONE AGAINST AEDES AEGYPTI!

Discard any objects you don’t use and that could accumulate water in the garbage.

Place the garbage in plastic bags and keep the garbage can properly covered with a lid on.

Add sand up to the edge in plants plates.

PREVENTION AND CONTROL.

**Salvador Poster 20 (S20)**

EVERYONE AGAINST AEDES AEGYPTI!

Bring your old tires to the urban garbage pick up service or store them empty of water in a covered location and away from the rain.

Keep the gutters clean.

Always store bottles upside down.

PREVENTION AND CONTROL.

**Salvador Poster 21 (S21)**

EVERYONE AGAINST AEDES AEGYPTI!

Keep the water tank closed.

Keep buckets and barrels closed with a lid on.

Wash water storing buckets with brush and soap on a weekly basis.

PREVENTION AND CONTROL.

**Salvador Poster 22 (S22)**

Backyard

Or you take responsibility or the mosquito takes over.

**Salvador Poster 23 (S23)**

Zika’s house.

Terrible neighbourhood for you.

Avoid stagnant water.

**Salvador Poster 24 (S24)**

UFBA AGAINST AEDES

You´re involved in this fight

Avoid stagnant water and eliminate potential mosquito breeding sites.

See how:

Keep buckets and barrels well covered with lids on.

Wash water storing buckets with brush and soap on a weekly basis.

Keep the water tank properly closed. Also add a mosquito net in the top of water tank (???).

Remove leaves, branches and anything that could possibly prevent water from draining through gutters.

Don’t let water accumulate on the “laje”

Fill plates of flower vases with sand up to the top.

Another option for the plates for plants is to wash them weekly.

Change the aquatic plants water and wash them with a brush, water and soap once a week.

Place the garbage in plastic bags and keep the garbage can properly closed.

Close the garbage bags properly and put them outside and out of reach of animals.

Store bottles upside down, avoiding water from accumulating inside them.

Tires must be fixed in covered locations.

Always maintain swimming pools and water fountains using adequate chemicals.

If your drain is not an open-close one, place a thin net in order to avoid the mosquito from reaching the water.

Place sand in all the broken glass bottles which could accumulate water.

Don’t leave accumulated water in dry leaves and bottle toppers.

Hardly used or non used toilets must be covered and checked on a weekly basis.

Always clean the air conditioning in order to avoid accumulating water.

Canvas used to cover objects or rubble must be tightened in order to avoid water puddles.

More information in [www.combateaoaedes.saude.gov.br](http://www.combateaoaedes.saude.gov.br)
